# Why Do They Do It? The Psychology Behind Antisocial Behavior in Children and Adolescents

**DOI:** 10.3390/pediatric17020026

**Published:** 2025-02-25

**Authors:** Marianna Mazza, Francesco Maria Lisci, Ester Maria Marzo, Valeria De Masi, Francesca Abate, Giuseppe Marano

**Affiliations:** 1 Unit of Psychiatry, Fondazione Policlinico Universitario A. Gemelli IRCCS, Largo Agostino Gemelli 8, 00168 Rome, Italy; fmlisci@gmail.com (F.M.L.); estermarzo7@gmail.com (E.M.M.); valeriademasi95@gmail.com (V.D.M.); abatefranci@gmail.com (F.A.); giuseppemaranogm@gmail.com (G.M.); 2 Department of Neurosciences, Università Cattolica del Sacro Cuore, 00168 Rome, Italy

**Keywords:** mental health, psychiatric disorders, antisocial personality disorder, violent behavior, forensic psychiatry

## Abstract

Antisocial Personality Disorder (ASPD) is a complex and often debilitating condition that can emerge from early behavioral disturbances in childhood and adolescence. This narrative review provides a comprehensive overview of the current understanding of ASPD in pediatric and adolescent populations, examining key diagnostic challenges, developmental trajectories, and emerging treatment approaches. Recent research underscores the critical role of the early identification of conduct disorder (CD) and oppositional defiant disorder (ODD) as precursors to ASPD. Specific attention is given to biological, environmental, and psychosocial risk factors, including genetic predispositions, family dynamics, and socio-economic adversity. Additionally, neuro-psychological research highlights deficits in executive function, emotion regulation, and social cognition, which may underline the persistent antisocial patterns. Neuroimaging studies suggest atypical neural activity in regions associated with empathy, reward processing, and impulse control. Effective intervention remains a challenge, as treatment options are limited and often complicated by co-occurring conditions, such as attention deficit hyperactivity disorder (ADHD) and mood disorders. Promising evidence supports the efficacy of integrative, multimodal approaches combining behavioral therapy, family interventions, and pharmacotherapy to reduce symptom severity and improve long-term outcomes. The review concludes by advocating for a public health approach that emphasizes prevention and early intervention, aiming to mitigate the progression to full ASPD in adulthood.

## 1. Introduction

Antisocial Personality Disorder (ASPD) is a complex, persistent disorder characterized by pervasive patterns of disregard for and violation of the rights of others. This includes behaviors such as deceitfulness, impulsivity, aggression, irritability, and a pronounced lack of remorse for harmful actions. ASPD often emerges from patterns of conduct disorder in childhood or adolescence, making early identification a key priority in mitigating its long-term impact. Individuals with ASPD frequently face challenges such as criminal behavior, substance abuse, interpersonal conflicts, and other social and occupational difficulties. According to Ref. [1], ASPD is a relatively common personality disorder, with estimates suggesting a prevalence of approximately 1–4% in the general population. The prevalence is notably higher in specific settings, particularly within forensic and incarcerated populations, where rates can reach 50–80%, underscoring its strong association with criminal and aggressive behaviors. Males are more frequently diagnosed with ASPD than females, with ratios often ranging from 3:1 to 5:1, reflecting both potential biological and social factors that influence its development [2], ref. [3]. It has been noticed that ASPD remains understudied in certain populations, particularly in adolescents, where antisocial behaviors may initially manifest but may not meet full diagnostic criteria until adulthood [3]. Early onset, typically during childhood or adolescence, is associated with more severe manifestations of the disorder and greater risk of co-occurring mental health conditions. Co-morbidities such as substance use disorders, depression, and anxiety are common in individuals with ASPD and may contribute to a more complex clinical presentation. The high prevalence of ASPD in high-risk populations, including incarcerated individuals and those with substance use disorders, prefigure significant public health implications [4].

The conceptualization of ASPD has evolved within the Diagnostic and Statistical Manual of Mental Disorders (DSM) over the years. In DSM-III, ASPD was defined as a disorder characterized by chronic antisocial behaviors that begin in childhood or adolescence. This diagnostic criterion required evidence of a Conduct Disorder before the age of 15, including persistent aggression, deceitfulness, rule-breaking, and a lack of empathy or remorse [5]. In DSM-III a particular emphasis has been placed on overt behavioral patterns rather than underlying personality traits, marking a shift from psychodynamic interpretations toward more observable, behaviorally defined criteria. The diagnosis of ASPD evolved significantly from DSM-III to DSM-IV, with changes focusing on diagnostic criteria, conceptual approach, and differentiation from psychopathy: the emphasis on psychopathy was reduced, making ASPD broader and behavior-focused [6]. In DSM-5, ASPD continues to be characterized by a pervasive pattern of disregard for and violation of the rights of others, beginning in childhood or early adolescence and continuing into adulthood. The DSM-5 criteria maintain the requirement of evidence of conduct disorder symptoms before the age of 15, specifying at least three of the following behaviors: repeated unlawful acts, deceitfulness, impulsivity, irritability and aggressiveness, reckless disregard for the safety of others, consistent irresponsibility, and lack of remorse. The DSM-5 criteria align with the DSM-III in its focus on overt behaviors, though it further outlines the continuity of antisocial behaviors from adolescence into adulthood [6]. This evolution in the DSM reflects ongoing debates about underlying mechanisms, including how ASPD could be better distinguished from other personality disorders, such as borderline and narcissistic personality disorders, and from psychopathy, a related but distinct construct not formally included in the DSM. In both DSM-III and DSM-5, however, diagnostic criteria of ASPD underscore the chronic, maladaptive patterns of behavior that severely impact personal, social, and occupational functioning. This diagnostic framework highlights the importance of early identification and intervention, given the foundation of this disorder is often laid during formative years, making childhood and adolescence crucial periods for effective prevention and treatment efforts.

Early identification of ASPD traits is essential due to profound personal and social consequences. Adolescents who display early antisocial behaviors, especially those combined with conduct disorder, are at heightened risk of developing full-blown ASPD. Interventions targeting younger populations could potentially reduce the likelihood of progression to ASPD in adulthood and lower the associated public health burden. By addressing factors such as family environment, peer influences, and early behavioral markers, mental health professionals may be able to implement prevention strategies that reduce or alter the course of these maladaptive behaviors [7]. Furthermore, early intervention could provide a window to address co-occurring conditions such as attention deficit hyperactivity disorder (ADHD) or depressive disorders, which are prevalent among youths with anti-social tendencies. Therapeutic interventions, especially when integrated with school- and community-based support systems, have shown promise in addressing the root causes of antisocial behaviors, offering affected individuals the opportunity to build more adaptive social and behavioral patterns [6].

## 2. Risk Factors and Social Influences on ASPD Development

The development of ASPD is shaped by a complex interplay between genetic and biological vulnerabilities and social influences. These factors interact dynamically to create a pathway that fosters antisocial behaviors, beginning in childhood and potentially culminating in full-blown ASPD in adulthood. Understanding these contributing elements can aid in identifying at-risk individuals and devising effective intervention strategies. Antisocial behavior refers to actions that violate social norms, disregard the rights of others, and often lead to harm. In youth, these behaviors range from rule-breaking and lying to aggression and theft, while in adults, they may evolve into criminality and a pervasive disregard for societal rules [1]. Conduct problems represent a spectrum of behaviors that disrupt societal norms and expectations, often clinically categorized as Oppositional Defiant Disorder (ODD) or Conduct Disorder (CD). CD can be considered as a robust precursor to ASPD, especially when its onset occurs before age 10 (childhood-onset type). Young subjects with CD frequently exhibit a chronic pattern of aggression, deceitfulness, and violation of rules, which, if un-treated, tend to persist and aggravate in adolescence and adulthood [8].

To better understand the importance of considering CD in the developmental trajectory of antisocial behavior, it is sufficient to consider that most adolescents admitted to rehabilitation institutions have a history of CD, and that about one-quarter of the adult prison population has a history of childhood psychiatric disorders [9,10,11]. Literature data indicate that between 1.5% and 5.5% of the juvenile population is affected by CD, and half of these individuals exhibit antisocial behavior in adulthood. Antisocial behavior appears to emerge in adulthood in only a smaller percentage of cases, ranging from 5% to 12% [12,13]. Early onset seems to be one of the most significant predictive factors for long-term continuity, prompting many authors to distinguish between early-onset and adolescent-onset ASPD. The first type involves a small group of male children and is characterized by a strong genetic component and a strong association with hyperactivity, limited cognitive and verbal skills, and impulsivity. The second type, with a later onset, is more influenced by environmental risk factors, particularly of a social nature [14].

The continuity between CD and ASPD is underpinned by shared genetic, neurobiological, and environmental risk factors, as suggested in the biopsychosocial model. This approach, introduced by George L. Engel in 1977 as a response to the limitations of the traditional biomedical model, recognizes the interdependence of biological, psychological, and social dimensions in shaping health and well-being, promoting a more integrated and person-centered approach to care [15]. In this scenario, the developmental trajectory of antisocial behavior should be viewed as the result of both traumatic and protective factors, of both genetic and environmental nature. While not all individuals with CD develop ASPD, early identification and intervention are critical in altering this evolution. Among the factors that can influence the developmental trajectory of antisocial behavior, a distinction can be made between risk factors and protective, or resilience, factors (Table 1). The first group includes risk factors, which can be further divided into individual factors, which may be organic and/or psychological, such as temperament, impulsive behavior, hyperactivity, substance use, social withdrawal, intellectual and neuropsychological difficulties, and family factors, which encompass disadvantaged socioeconomic conditions, low levels of education within the family, the presence of antisocial behaviors or psychopathology in parents, abuse and/or neglect, and substance use. In addition, school and peer group may play a significant role: school-related factors include poor academic performance, lack of interest and motivation, and inefficiency of educational institutions, while peer group factors involve association with peers who exhibit deviant or violent tendencies, or isolation due to peer rejection [12,16,17,18]. Childhood abuse, including physical, emotional, and sexual maltreatment, has been associated with the development of CD and ASPD. Adverse childhood experiences, such as neglect and exposure to domestic violence, disrupt normal emotional and cognitive development, increasing the likelihood of externalizing behaviors, aggression, and impulsivity [19]. Studies have shown that individuals who experience abuse at an early age often develop maladaptive coping mechanisms, including hostility, deceitfulness, and rule-breaking, which are hallmark features of CD and may persist into adulthood as ASPD [19,20,21,22]. Furthermore, exposure to chronic abuse can disrupt attachment processes, leading to deficits in empathy and increased risk for callous/unemotional traits, and leading to further sexual victimization through association with sexually abusive peers or involvement in dangerous situations or sexual survival strategies [21,22]. The second group includes protective or resilience factors, which affect the individual’s ability to cope with adversity by leveraging both personal and environmental resources (family, school, community). These involve endogenous factors, such as behavioral inhibition and harm avoidance (characterized by apprehension, anxiety, and shyness) and behavioral activation, which is linked to novelty and sensation-seeking. High levels of behavioral activation and low levels of behavioral inhibition are strongly associated with the development of antisocial tendencies. Stable and strong bonds with the family of origin and supportive institutions (school or community) that can assist the individual during challenging times may be considered as exogenous protective factors [23].

This combination of risk factors should be seen as part of a dynamic process that begins in early childhood and continues into adulthood. It is important to note that the antisocial development observed in adolescence is often, though not always, preceded by difficulties and antisocial traits in childhood. This highlights the need for clinicians to pay particular attention to the development of CDs and to the presence of phase-specific factors that can influence the developmental trajectory at any point. For instance, children who did not display antisocial tendencies in preadolescence may engage in deviant and violent behaviors during adolescence, ranging from common offenses to more severe crimes such as rape or murder. At any stage of psychosocial development, events can occur that may shape a deviant evolution [24]. From a developmental perspective, these adolescents often exhibit trouble in taking responsibility for their behaviors, attentional deficits, problems in managing impulses and, in general, difficulties in internalizing control and mentalizing the consequences of their actions [25]. ASPD has indeed been placed at the extreme end of the narcissistic personality disorder spectrum, with two key factors defining its psychopathological features: aggressive narcissism and an antisocial lifestyle [26]. Aggressive narcissism is characterized by egocentrism, insensitivity, and a lack of remorse or guilt, while the antisocial lifestyle is marked by irresponsibility, impulsivity, and a constant pursuit of thrilling situations. A precipitating factor is often a difficulty with symbolization, associated with low socio-economic status, limited intelligence, and poor educational attainment. The antisocial trajectory can thus be divided into two pathways: one characterized by impulsive aggression, involving reactive aggression (responsible for more common offenses and representing classical antisociality), and the other by sadistic–predatory aggression (responsible for more severe crimes and defining classical psychopathy) [27,28].

## 3. Neurobiological Bases of Violent Behavior

Genetics and environmental factors synergistically contribute to antisocial behaviors. Extensive investigation has been conducted into hormonal factors, implicated neurotransmitters, perinatal risk factors, and neuroimaging studies, to explain the aggression and impulsivity that, among other traits, characterize antisocial individuals [29].

### 3.1. Gender Differences and Hormonal Role

Although the prevalence of ASPD is approximately 3:1 in men compared to women, there is limited research exploring sex differences in the correlates of ASPD within the general population. Studies have highlighted a difference in the severity and type of antisocial behavior between sexes. Men are more frequently involved in criminal and aggressive actions, while women with conduct disorders tend to display antisocial behavior in forms such as bullying, engaging in promiscuous and unprotected sexual activity, substance abuse, and running away from home [30]. During childhood, antisocial behavior is observable in ODD and CD, both of which fall under the category of Disruptive Behavior Disorder (DBD). While both disorders involve defiant behavior, conduct disorder is marked by more severe actions, such as aggression toward people and animals, property destruction, and serious rule violations that can lead to legal consequences. In contrast, ODD primarily involves defiance towards authority figures without the same level of severity [8,31]. Various studies have been conducted on the role of oxytocin and antisociality, focusing on oxytocin receptor gene (OXTR) variant rs1042778. Oxytocin plays a role in the recognition of facial emotions such as happiness and fear, which is essential for empathy and interpersonal relationships. The genotype TT rs1042778 has been shown to be associated with a lack of guilt and empathy and emotional constrictedness, which are typical features of DBD. This study showed that in men, but not in women, the OXTR rs1042778 TT genotype was associated with increased right amygdala reactivity to angry facial expressions, which was uniquely associated with higher levels of antisocial behavior in men [32]. In addition, children and adolescents with more severe DBD diagnoses exhibit higher methylation of the OXTR gene and lower endogenous oxytocin levels. Unfortunately, this study did not include the female population [33].

A meta-analysis examined the association between eight single nucleotide polymorphisms (SNPs) in the OXTR and antisocial behavior, analyzing data from 15 samples in 12 studies with a total of 12,236 participants. Since the findings were accompanied by high heterogeneity and concerns about quality control, authors highlight the need for caution in interpreting previous findings and underline the need for further research to clarify the role of OXTR in antisocial behavior [34]. A recent study found that intranasal oxytocin reduced right amygdala hyperactivity to angry faces in ASPD participants, bringing their amygdala activity to levels comparable to healthy controls. The effects of oxytocin were more pronounced in women with ASPD, suggesting that oxytocin may have therapeutic potential for the treatment of reactive aggression, particularly in female patients [35].

Regarding the correlation between testosterone and human aggressiveness, studies have demonstrated increased testosterone concentrations during competitive acts, predicting ongoing and future aggressiveness. Testosterone is significant for its effects on the brain from embryonic stages. Beginning early in fetal development, the androgen receptor gene helps regulate bodily exposure to testosterone. Most studies of individuals have found an inverse correlation between the number of CAG repeats on this gene and serious forms of physical aggression [36].

In a study on 545 prisoners, a division was made into four groups with different psychological, biological and criminal characteristics, the first comprising subjects with intermediate scores in the categories used, the second characterized by subjects with few comorbidities, and the last two with persons with personality disorders (PDs) and substance use disorders (SUDs). When analyzing the levels of various biological markers, it was noted that testosterone levels were higher in the group with younger inmates and more severe psychiatric symptoms. It was also noted that cortisol levels were lower in the groups with PDs and SUDs while they were higher in the second group [37]. Another study showed increased levels of testosterone and sex hormone-binding globulin (SHBG) in inmates, correlating these findings with increased aggression. High levels of SHBG also correlated with an increased likelihood of APSD in recidivist patients [38].

Neuroimaging techniques have shown that in adult humans, this hormone stimulates the amygdala, enhancing the intensity of emotional responses and reducing the ability of the prefrontal cortex to regulate impulsive behaviors and regulate aggressive responses. Conversely, cortisol and serotonin (via inhibitory receptors) reduce the effects of testosterone, limiting aggression and impulsiveness. Cortisol, in particular, reduces impulsive tendencies from the prefrontal area into the subcortical structures. The presence of testosterone therefore stimulates the amygdala, inducing more impulsive and aggressive behavior [39]. Impulsivity and aggressiveness are the characteristics that make emotional responses inappropriate and exaggerated in frustrating or threatening contexts [40].

The nesfatin-1 hormone is a hypothalamus hormone that plays a role in nutrition and maintaining the body’s energy balance. Additionally, it has been reported to play a role in the regulation of stress response of the central nervous system. Nesfatin-1 hormone levels in APDS patients have been found to be lower compared to those of the healthy controls [41].

Leptin and ghrelin hormones play a significant role in response to stress and relate to numerous psychiatric disorders. High levels of ghrelin and low levels of leptin have been observed in patients diagnosed with ASPD [42].

### 3.2. Neurotransmitters

The regulation of emotional responses and aggression is mediated by neurotransmitters such as serotonin, dopamine, and acetylcholine, as well as by interactions between subcortical brain structures, such as the amygdala and hypothalamus, and prefrontal areas. Neurotransmitters are essential in regulating emotions, behaviors, and social interactions. Disruptions in these systems may contribute to the impulsivity, aggression, and emotional dysregulation seen in individuals with ASPD. For example, dopaminergic deficiency and an increase in serotonergic drive or an imbalance among testosterone, cortisol, and serotonin can increase aggression, leading to reduced impulse control through their respective neural circuits [43].

Regarding acetylcholine, experiments using agonists and antagonists of cholinergic receptors have demonstrated that stimulation of the cholinergic system is correlated with aggression, with variations in the type of aggression depending on which brain area is stimulated [44,45].

Serotonin is one of the most widely studied neurotransmitters in relation to aggression, impulsivity, and mood regulation. It plays a central role in modulating emotions, controlling impulses, and maintaining social behavior. Serotonin plays an essential role in inhibiting the prefrontal cortex: low serotonin levels can be associated with more aggressive behavior, whereas high levels correlate with greater inhibition of such behaviors [46]. Several studies have demonstrated that individuals with ASPD often exhibit serotonin dysregulation. Studies in rats have shown that serotonin depletion resulted in aggressive behavior [47], whereas serotonin-mimetic drugs had an inhibitory effect on aggressive behavior [48].

Dysfunction of the serotonergic system affects the individual but cannot preempt social skills, impulse control, and capacity for emotional regulation [49]. A study of serotonin metabolites showed a correlation between aggression and low levels of 5-hydroxyindoleacetic acid (5HIAA) [50]. It was also evidenced that aggressive behavior or suicide attempts are linked to low serotonin levels [51].

It is noteworthy that serotonin levels are generally higher in females compared to males. The polymorphic variants of genes involved in the synthesis, release, and elimination of serotonin are therefore crucial. Specifically, polymorphisms of the promoter coding for the enzyme monoamine oxidase A (MAO-A), which is involved in the metabolism of various neurotransmitters, including dopamine and serotonin, have been studied. A statistically significant correlation has been found between low MAO-A activity and antisocial tendencies in abused children [52].

A study by Coccaro et al. [53] found that low cerebrospinal fluid (CSF) levels of 5-hydroxyindoleacetic acid (5-HIAA), a metabolite of serotonin, were associated with aggressive behavior in individuals diagnosed with personality disorders, including ASPD. Similarly, a meta-analysis by Alvarez et al. [54] highlighted that serotonin dysfunction was linked to impulsive and violent behaviors in individuals with ASPD.

Serotonin’s role in aggression has been further elucidated through research on the serotonin transporter gene (5-HTTLPR). Variants of this gene have been associated with increased susceptibility to impulsive aggression. A study by Hariri et al. (2005) showed that individuals with the short allele of the 5-HTTLPR gene exhibited higher levels of aggression, particularly in response to environmental stressors [55].

Dopamine, another critical neurotransmitter, is implicated in reward processing, motivation, and the regulation of pleasure and pain. Dysregulation of the dopaminergic system has been strongly linked to impulsivity, risk-taking behaviors, and a reduced response to punishment—behaviors commonly observed in individuals with ASPD. Research suggests that individuals with ASPD may exhibit an altered dopaminergic system, particularly in areas of the brain involved in reward and motivation, such as the ventral striatum and prefrontal cortex: individuals with ASPD may have elevated dopamine release in response to rewarding stimuli, which could contribute to their tendency to seek out high-risk or reward-based activities, even at the expense of others [56,57,58]. The role of dopamine in ASPD is further supported by studies examining the relationship between dopaminergic genes and antisocial behaviors. For example, a study by Lukkarinen et al. found that genetic variations in the dopamine D2 receptor were associated with a higher risk of developing psychopathic traits [59]. Additionally, individuals with ASPD often show deficits in the dopaminergic regulation of the prefrontal cortex, an area of the brain involved in impulse control, moral decision-making, and behavioral inhibition [60,61].

Gamma-aminobutyric acid (GABA) is the principal inhibitory neurotransmitter in the central nervous system. It plays a crucial role in regulating neural excitability and maintaining a balance between excitation and inhibition in the brain. Reduced GABAergic activity has been linked to heightened aggression, impulsivity, and poor emotional regulation, traits frequently associated with ASPD. Research examining GABA’s role in ASPD suggests that lower GABA levels may contribute to the emotional dysregulation and impulsivity seen in individuals with the disorder. A study by Denson et al. found that low GABA levels in the prefrontal cortex were associated with increased aggression and impulsivity. The prefrontal cortex is essential for higher-order cognitive functions, including impulse control, decision-making, and moral reasoning. Disruptions in GABAergic signaling in this region may lead to difficulty in regulating aggressive impulses and engaging in prosocial behavior [62,63].

Furthermore, neuroimaging studies have shown that individuals with ASPD often exhibit abnormalities in the functioning of brain regions that are rich in GABA receptors, such as the amygdala and prefrontal cortex. These regions are critical for processing emotional responses and regulating social behavior. Dysfunction in GABAergic systems may impair the ability to inhibit aggressive impulses, leading to the antisocial behaviors characteristic of the disorder [62].

While these findings have provided important insights into the neurobiological mechanisms underlying the disorder, further research is needed to fully elucidate the complex interactions between neurotransmitter systems and environmental factors in the development of ASPD.

### 3.3. Perinatal Factors

Recent studies have highlighted that a significant portion of the factors influencing the development of aggressive and violent behaviors originate from the family environment. Through genetic and epigenetic factors, the individual’s vulnerability to such behaviors is influenced from the embryonic stage, integrating both biological and social aspects [64]. There is evidence that perinatal factors—events and exposures during pregnancy and around the time of birth—may play a critical role in the development of APSD. The perinatal period is a crucial window for brain development and can significantly influence long-term psychological and behavioral outcomes.

Maternal mental health conditions during pregnancy, particularly depression and anxiety, have been linked to behavioral problems in children, including aggression and antisocial behaviors. Maternal depression during pregnancy is associated with alterations in fetal brain development, particularly in regions involved in emotional regulation and social behavior, such as the prefrontal cortex and amygdala. These alterations can increase the risk of externalizing behaviors, such as aggression and impulsivity, which are characteristic of ASPD [65]. Furthermore, maternal malnutrition during pregnancy has been identified as a risk factor for developmental disorders. Inadequate prenatal nutrition, especially a lack of essential vitamins and minerals, can disrupt neurodevelopment and lead to cognitive and emotional dysregulation in offspring. These children are at a higher risk of developing personality disorders, including ASPD, in later life [66].

Exposure to prenatal stress is associated with an increased risk of aggression, impulsivity, and other externalizing behaviors. A study by Van den [67] found that prenatal stress exposure was linked to an increased risk of conduct disorder and aggression in children. These children may be more likely to develop ASPD later in life, as early stress exposure can lead to dysregulation of the hypothalamic–pituitary–adrenal (HPA) axis, a key system involved in stress and emotional regulation [67].

Retrospective research has linked aggressive behavior, particularly in individuals with a higher predisposition to antisocial behaviors, with the use of alcohol, tobacco, and drugs during pregnancy, complications during pregnancy and childbirth, and maternal rejection. Stressful factors such as disadvantaged socioeconomic conditions or conflicts can also contribute to a biological pre-disposition, promoting inadequate parenting behaviors [68]. Among substances, maternal smoking during pregnancy is associated with an increased risk of developing antisocial behaviors later in life [69]. Studies have shown connections between smoking and behavioral disorders in adolescence, both violent and non-violent crimes, and antisocial tendencies. The biological mechanisms involved may include oxygen deprivation and alterations in neurochemical systems involving norepinephrine, serotonin, and dopamine [70].

Birth complications, such as low birth weight, preterm birth, and oxygen deprivation during delivery, have also been linked to an increased risk of developing ASPD. These complications can lead to brain injury or developmental delays, particularly in areas of the brain involved in emotional regulation, impulse control, and decision-making [71,72].

### 3.4. Neuroimaging Studies

Behavioral disorders associated with psychopathy are strongly linked to dysfunctions in specific brain areas that are part of neural networks involved in emotion processing and behavior control. These networks include connections between the orbitofrontal area and the limbic system, which are involved in emotional processing, somatic reactions to emotional stimuli, action planning, and decision-making. Additionally, the connection between the anterior cingulate and the orbitofrontal area is important for the correct attribution of emotional value to social stimuli and impacts violent, aggressive, or defiant behavior [73]. Another crucial network includes the prefrontal, temporal, and limbic areas, which are involved in processing and responding to emotional stimuli. Brain areas most affected in these disorders include the insula, temporal cortex (especially the superior temporal gyrus), anterior cingulate, inferior frontal cortex, orbitofrontal area, dorsal and ventral regions of the prefrontal cortex, amygdala, ventral striatum, and other basal ganglia [74].

Psychopathy is characterized by impairments in emotion processing and learning, as well as social and emotional decision-making functions. These difficulties are closely related to specific neurological changes that regulate these functions. Aggressive reactions to interpersonal threats or provocations may be explained by a common factor: anger and hostility, which are linked to dysfunctions between prefrontal areas and subcortical structures, particularly the amygdala. Moreover, in borderline personality disorder (BPD) and ASPD, alterations in the frontal, temporal, and limbic areas and in the serotonergic and endocannabinoid systems may explain the connection between impulsivity and aggression. Reduced connectivity between the cortex and the striatum may be associated with a higher predisposition to risky and violent behavior [75].

Through neuroimaging studies, initially conducted using EEG and later expanded with the use of imaging techniques such as computed tomography (CT), magnetic resonance imaging (MRI), functional MRI (fMRI), and positron emission tomography (PET), the importance of the limbic system in modulating aggressive behavior has been demonstrated. Amygdala dysfunction has been associated with psychopathy, particularly regarding its role in aversive conditioning, instrumental learning, and response to emotions such as fear and sadness. Individuals with high psychopathy scores have shown a reduced amygdala volume [76]. The hypothalamus also plays a role in modulating aggression, and lesions in the frontal lobe, prefrontal cortex, and temporal lobe have been linked to aggressive behavior [77]. Dysfunction in the amygdala and prefrontal cortex has also been associated with emotional dysregulation, with a diminished or absent capacity to feel guilt and remorse following aggressive actions. The autonomic nervous system also plays a role: it is protective in individuals with antisocial disorders who do not have alterations. Hypoactivity in this system would lead to deficiencies in learning from the social consequences of risky and aggressive behavior [78].

Studies have shown early alterations in the functioning of the hypothalamic–pituitary–adrenal (HPA) axis and in the serotonergic system, with hypoactivity correlating with a predisposition to antisocial behavior. Stress and adverse childhood events may play a role in the problematic functioning of these systems [79]. It has been hypothesized that early adverse experiences play a crucial role in the development of chronic antisocial behavior in children with low callous/unemotional traits and HPA axis hyperactivity. Callous/unemotional traits refer to a cluster of characteristics such as low empathy, lack of remorse, and insensitivity to the emotions of others, delineating a group of youth at high risk for severe antisocial behavior. In children with high callous/unemotional traits and HPA axis hypoactivity, a particularly severe subgroup has been observed where antisocial behavior tends to develop relatively independently from adverse experiences [80].

## 4. Psychological Aspects of ASPD

Psychological research on ASPD has revealed significant impairments in cognitive and emotional regulation, empathy, and impulsivity that contribute to characteristic behavioral patterns observed in individuals with the disorder. These dysfunctions have profound implications for understanding the disorder and for the development of therapeutic approaches aimed at addressing its complex psychological dimensions.

### 4.1. Cognitive and Emotional Dysregulation

Neuropsychological impairments commonly associated with CD include low Intelligence Quotient (IQ), language deficits, and executive function (EF) impairments [31,81]. These deficits are often accompanied by atypical social cognition, characterized by distorted processing of social information. Low IQ has been consistently linked to CD, even when controlling socioeconomic and demographic factors [82]. This association is particularly pronounced in individuals with early-onset CD [83]. Language deficits are another prominent feature of CD, persisting independently of IQ or sociodemographic variables [84]. A notable finding is the discrepancy between Performance IQ (PIQ) and Verbal IQ (VIQ), reflecting specific impairments in verbal abilities. These deficits impact several domains, including verbal comprehension, production, memory, reading, writing, and verbal problem-solving [85]. Theoretical and clinical models link these verbal impairments to antisocial and aggressive behavior and researchers propose that deficits in verbal memory and abstract reasoning hinder self-regulation and impair early socialization processes [27]. The critical role of internalized language in developing self-regulatory functions has been emphasized, alongside EF components such as behavioral inhibition, working memory, emotional and motivational regulation, and behavioral analysis and synthesis. Additionally, verbal deficits may disrupt conditioning processes. Children with language impairments often struggle to encode behaviors using verbal labels like “bad” or “incorrect”, relying instead on trial-and-error learning. Consequently, they experience punishment more frequently than their peers, yet with limited success in reducing problematic behaviors due to the lack of robust verbal mediation [77].

Executive functions, essential for self-regulation and goal-directed behavior, are also frequently impaired in individuals with ASPD and in children with CD. Pennington and Ozonoff conducted a foundational review of EF studies across four developmental psychopathologies including ADHD, CD, and Tourette syndrome. They found that significant EF deficits were evident in autism and ADHD but were not consistently observed in “CD without associated ADHD” or in Tourette syndrome [86]. These authors reviewed nine studies in patients with CD, conducted between 1988 and 1996, which did not distinguish subjects based on age of onset. The findings showed significant EF impairments in individuals with CD [87], but only when ADHD comorbidity was present. In cases of CD without ADHD, no EF deficits were observed. However, subsequent studies in both general population samples and clinical populations have challenged these conclusions [88]. A correlation has also been found between EF deficits and conduct disorders, even in the absence of ADHD, suggesting a broader relationship between EF impairments and CD. Further research supports a link between EF impairments and criminal offending [89,90]. These impairments appear heterogeneous and may be influenced by factors as reported in [89]: individuals with ASPD and low psychopathic traits display heightened attentional impulsivity, whereas those with high psychopathic traits do not [91,92].

Studies employing Stroop tasks (measure of cognitive interference and the ability to manage response inhibition) suggest subtle deficits in cognitive control among antisocial individuals, but inconsistent findings point to the influence of sample characteristics and task methodologies [93,94]. Additionally, many studies show that impairments in cognitive regulation may not universally apply across individuals with ASPD but are influenced by the presence and extent of psychopathic traits, highlighting the complexity of cognitive dysregulation within this population [95,96]. The psychopathic personality style reflects a constellation of traits, including remorselessness, callousness, deceitfulness, ego-centricity, a lack of interpersonal bonds, superficial charm, externalization of blame, and a lack of fear and anxiety [97].

Emotion regulation is integral in the development and maintenance of mental health, and it has been theorized that problematic emotion regulation underlies many features of psychopathology [98]. A growing body of research has provided evidence of emotion regulation problems in people who engage in aggression and violent behaviors, including individuals with psychopathic traits [99,100]. In a study by Casey and colleagues, individuals with high psychopathy exhibited an amplified cardiovascular response when processing negative emotional images compared to positive ones. This may indicate that, for highly psychopathic individuals, typically unpleasant material holds an anomalously rewarding quality [101]. Furthermore, when participants were instructed to attempt to experience an emotional response by “getting into the feeling” of the emotion conveyed by the image, those with the highest levels of psychopathy demonstrated reduced responsiveness. This suggests that they were less capable of engaging emotionally with the content. Maladaptive emotion regulation occurs when the emotional response is not changed in the desired way, the long-term costs of the emotional response outweigh the short-term influences on emotion, strategies are applied in a rigid manner inconsistent with long-term goals, or attempts at emotion suppression or resistance result in maladaptive secondary emotional responses [102]. Emotion regulation is the automatic or controlled manipulation of the presence and/or intensity of the components of an emotional response-including subjective experience, physiological activity, or behavior [102]. The term emotion dysregulation broadly refers to failures in regulation strategies as well as deficits or impairments that contribute to difficulties in managing emotional states. Within the emotion regulation literature, two dominant perspectives outline the relationship between emotion regulation and psychopathology [100]. The deficit emotion regulation (DER) perspective focuses on specific impairments in psychological functioning that result in a reduced ability to regulate emotions effectively. From this viewpoint, some individuals regulate their emotions less frequently or effectively than others. In contrast, the maladaptive emotion regulation (MER) perspective highlights the use of specific, atypical strategies to modulate emotional states. This perspective assumes that healthy emotion regulation encompasses several processes that operate differently throughout emotion generation [98]. For example, antecedent-focused cognitive strategies occur before the emotional response and aim to select or modify the situation or reinterpret its meaning. Conversely, response-focused strategies are employed after the emotional response is activated, directly impacting the emotional experience itself. Emotion regulation strategies can also vary in their level of awareness. Some are intentionally and explicitly chosen to manage emotions, while others are applied implicitly, without conscious awareness of their effect. Additionally, these strategies differ in their methods of influence and the goals they seek to achieve. Moreover, it is crucial to recognize that the psychopathic population is highly heterogeneous in terms of emotional regulation, emotion recognition, and overall emotional functioning. Research suggests that the ability to regulate emotions depends not only on the type of emotion being processed but also on the specific subtype of psychopathy [103]. High-functioning psychopaths often demonstrate greater emotional competence, enabling them to manipulate social situations more effectively, whereas lower-functioning psychopaths may exhibit more pronounced deficits in emotional processing and regulation. This differentiation influences their behavioral patterns, impulsivity, and responsiveness to interventions. Understanding these variations is essential when evaluating emotional dysregulation in psychopathy, as it highlights the complex interplay between cognitive and affective processes in antisocial behavior [103].

Considering these distinctions, emotion regulation strategies can be categorized based on the type of regulation (cognitive or response-focused), the level of awareness (implicit or explicit), and the goal or target of change (e.g., goal, context). This multidimensional framework underscores the complexity and versatility of emotion regulation as a construct. A 2021 study by Garofalo et al. [102] examined the potential role of emotional dysregulation in aggression among psychopathic individuals. The researchers investigated the indirect effects of psychopathy, mediated by emotional dysregulation, on various components of trait aggressiveness (anger, hostility, physical, and verbal aggression) as well as on different forms of aggressive behavior (reactive and proactive aggression). The findings revealed that emotional dysregulation explained the association between psychopathic traits and both reactive and proactive aggression. While the effect sizes were generally small, they were still statistically significant and clinically meaningful. As expected, the indirect effect on reactive aggression was more than twice as strong as that on proactive aggression. These results suggest that emotional dysregulation plays an important, albeit not exclusive, role in explaining the links between psychopathy and aggression. The connections between emotional dysregulation and “cold-blooded” correlates, such as the cognitive component of trait aggression (hostility), proactive forms of aggression, and psychopathic traits, also encompass a lack of empathy and callousness.

Based on these findings, alongside the current results, targeting emotional dysregulation in its various aspects appears to be a promising strategy for addressing several components and forms of aggression, even among individuals with psychopathic traits. Consequently, interventions focused on improving emotional awareness, acceptance, and behavioral control may be effective treatment strategies. It is likely that such interventions could be more effective if they also address critical risk factors for aggression, such as cognitive skills and empathy.

### 4.2. Empathy Deficits and Theory of Mind

Empathy, defined as the ability to access and respond to another person’s inner world, is a multidimensional construct involving cognitive, emotional, and self-regulatory mechanisms [104]. A lack of empathy has been strongly associated with antisocial behavior [105]. Research has demonstrated robust positive associations between parent–child and peer relationship quality and empathy in adolescence, implying that good empathic abilities may be a protective factor for experiencing poor relationships. Empathy seems therefore positively related to parental warmth [106]. The development of empathy is fundamentally influenced by attachment; in fact, secure attachment has a positive correlation with empathy, while avoidant attachment correlates negatively. It is still unknown how anxious–ambivalent attachment and empathy are related each other [107]. In psychopathic individuals, empathy deficits have been consistently reported and are thought to stem from reduced responsiveness to distress of other individuals, which diminishes the aversive nature of aggressive acts and makes them more likely to occur [108,109,110].

Theory of mind (ToM) is closely associated with empathy. It refers to the ability to understand and infer other people’s thoughts, feelings, intentions, and beliefs and is essential for successful social interaction. It can be categorized as social–perceptual ToM, which involves automatic recognition of mental states from facial expressions, and social–cognitive ToM, which entails reasoning about others’ mental states in more complex scenarios. Another distinction divides ToM into cognitive ToM, which allows for the inference of others’ thoughts, intentions, and beliefs, and affective ToM, which facilitates understanding their feelings and emotions [111]. During development, adolescence represents a critical period for ToM maturation, as substantial structural and functional changes in the brain enable individuals to navigate increasingly complex social environments [112,113]. Factors such as age, verbal IQ, and clinical conditions influence ToM abilities during this stage [114,115].

In people with psychopathy, ToM impairments are commonly observed, particularly in the recognition of others’ affective states, a function associated with affective ToM. However, there are less severe deficits than those found in conditions such as schizophrenia or autism [116]. Psychopathy’s characteristic manipulative and deceitful interpersonal style may be partly explained by specific ToM abilities, with some evidence suggesting that superior affective ToM performance is associated with increased premeditated aggression [117]. This implies that even though psychopaths lack emotional empathy, they may use affective ToM to manipulate others for their own benefit.

The study of ToM impairments in psychopathy allows important insights into how these individuals understand and respond to others’ mental states. Such research is critical for studying the risk of aggression and violence, particularly in youth with conduct problems. Psychopathy-related ToM deficits, especially in affective domains, highlight the intricate interaction between cognitive and emotional processing in this population.

Empathy and ToM are closely interconnected in development and social functioning, and a deficit in these areas contributes significantly to the challenges faced by individuals with ASPD. For this reason, it is important to deepen the nuances of psychopathy and its implications for social interaction and behavior.

### 4.3. Impulse Control and Reward Sensitivity

Impulsivity traits are defined as a predisposition to rapid, unplanned reactions to internal or external stimuli without considering the potential negative consequences of these reactions for oneself and others [118].

Numerous intrapersonal and interpersonal problems, including violent crimes, other illegal acts, repeated lying, gambling problems, unemployment, and promiscuity, can result from impulsive behaviors linked to ASPD, which frequently start in childhood or early adolescence [119,120]. Furthermore, as previously discussed, individuals with ASPD frequently engage in aggressive behaviors. Factor analyses have demonstrated that aggression can be categorized into two distinct forms: reactive aggression, which refers to behaviors triggered by provocation or frustration, and proactive aggression, which encompasses actions performed without accompanying emotions and primarily aimed at achieving specific personal goals [121,122].

The DSM-5 alternative model for personality disorders places a stronger emphasis on personality traits, requiring that at least six of the following pathological traits be present to diagnose ASPD: manipulativeness, callousness, deceitfulness, hostility, risk-taking, impulsivity, and irresponsibility [6]. In this framework, impulsivity is specifically defined as acting on the spur of the moment in response to immediate stimuli, acting without a plan or consideration of consequences, and having difficulty establishing and adhering to plan. Identifying the subcomponents of self-reported impulsivity and behaviorally measured impulse control most frequently affected in ASPD could enhance understanding of the disorder’s etiology, as well as improve diagnostic reliability and validity.

Previous studies on impulsivity in ASPD patients have produced mixed results. Research utilizing impulsivity questionnaires has consistently shown increased rates of trait impulsivity in ASPD patients compared to healthy controls (HCs) and other psychiatric patients [92,123]. However, findings from behavioral laboratory measures of impulsivity have been less consistent [92,124].

In a study by Mann and colleagues [125], using a large, nationally representative sample of youth followed from early childhood through mid-adolescence, researchers examined whether childhood conduct problems and low levels of guilt predicted age-related changes in disinhibited personality traits (i.e., impulsivity and sensation seeking) during adolescence. They also investigated whether these childhood characteristics moderated the association between changes in disinhibited personality traits and the development of antisocial behavior. The findings indicated that childhood problems and lack of guilt differentially predict initial levels of disinhibited personality traits in adolescence. Conduct problems were associated with higher levels of sensation seeking and impulsivity. Controlling for biological sex and conduct problems, low levels of guilt predicted lower levels of sensation seeking, defined as a strong need for stimulation, a low tolerance to boredom, and a willingness to take risks for the sake of having novel and varied experiences.

Childhood conduct problems and lack of guilt do not seem to significantly predict changes in disinhibited personality traits or antisocial behavior during adolescence. It has been reported that individuals with a history of childhood conduct problems and low levels of guilt do not exhibit more rapid increases in sensation seeking or slower decreases in impulsivity over time [125]. Behavioral research has long established that altered reward-related behavior plays a critical role in the development and persistence of antisocial behavior and its associated emotional and personality features [126].

More recently, researchers have been able to investigate the brain mechanisms underlying antisocial behavior and psychopathy thanks to neuroimaging techniques like fMRI. The persistence of antisocial behavior may also be linked to deficits in responding to, and learning from, both reward and loss. For example, people with chronic antisocial behavior often continue risky or illegal behaviors despite the likelihood of severe punishment, such as incarceration. This suggests that the neural systems governing reward valuation, loss processing, and learning may be impaired in these individuals [126].

Much of the research on the neural mechanisms of reward processing in antisocial behavior has focused on adolescents and underlines the complexity of the relationship between antisocial behavior and neural responses to reward. Some studies report frontostriatal hypersensitivity in individuals with high rates of reward system activation, while others find frontostriatal hyposensitivity [127,128]. Significant methodological heterogeneity, such as variations in the kinds of reward or loss paradigms, could be the cause of this discrepancy. Complex reinforcement behaviors, such as risky decision-making and delay discounting, involve multiple processes—such as risk/reward valuation, regulation, anticipation, and consumption—making it challenging to pinpoint the exact source of dysfunction [129]. Establishing connections between personality traits and fundamental reward-related processes is a critical step before examining more complex forms of reward processing. Furthermore, the frontostriatal circuitry undergoes rapid developmental changes during adolescence, which may limit the applicability of adolescent findings to adult populations [129].

Studying antisocial behavior in adults is fundamental because brain and behavioral traits, such as impulsivity and risk-taking, are more stable during adulthood [130]. Moreover, research on adults helps identify individuals with the most severe and chronic trajectories of antisocial behavior [131]. A systematic review by Murray and colleagues examines the links between antisocial behavior, psychopathy, and neural dysfunction in reward and loss processing, highlighting significant gaps in the existing literature. The findings suggest that antisocial behavior and psychopathic traits are associated with abnormal neural activity in regions such as the ventral striatum (VS), in particular during reward anticipation [132]. Few studies have investigated how different phases (e.g., anticipation vs. outcome) and valences (reward vs. loss) interact, leaving a significant gap in understanding. Additionally, research on loss processing remains notably underrepresented, despite evidence showing that individuals with psychopathic traits often fail to adapt from reward-focused strategies after punishment [133].

Other studies examining socially contextualized reward and loss tasks (e.g., delivering shocks or responding to unfair monetary offers) underline the complexity of these processes but also challenge the comparability of findings. Instead of focusing on discrete mechanisms of reward or loss, these activities might represent dysfunctions in learning, social interactions, and emotional processing. Therefore, it is important to develop paradigms that simultaneously address reward and loss across distinct phases to clarify the underlying neural and behavioral patterns [132].

### 4.4. Clinical Presentation and Comorbid Psychiatric Conditions

A study by Goodwin and Hamilton [134] explores the strong association between anxiety disorders and ASPD, showing that over half of people with ASPD have experienced at least one anxiety disorder in their lifetime. This co-occurrence is associated with a significantly higher risk of depression, substance dependence, and suicidal behavior, with the odds of attempting suicide nearly doubling when both conditions are present. Given that the direct correlation between ASPD and depression is diminished when comorbid anxiety and drug use disorders are considered, the study proposes that anxiety may act as a mediator in the interaction between these two illnesses.

In particular, a strong correlation has been demonstrated between ASPD and particular anxiety disorders, such post-traumatic stress disorder (PTSD). Depression is less common in adults with ASPD who do not experience anxiety, which is in line with the theory that ASPD is characterized by emotionless, frigid characteristics. Adolescents with anxiety, particularly those with specific phobias, may also be shielded from the onset of ASPD by behavioral disorders. Anxiety plays a significant role in aggravating the adverse consequences of ASPD and underscore the necessity of focused interventions that tackle these interrelated problems in order to lower risks like substance abuse and suicidality [134,135].

Numerous studies in the literature show a frequent comorbidity between ASPD and ADHD, a common developmental disorder characterized by symptoms of inattention, hyperactivity and impulsivity, with symptoms persisting into adulthood in more than 50% of cases [136,137]. Beyond these primary symptoms, people with ADHD frequently struggle with organization and emotional control, which can affect their ability to function in daily life and in relationships with others [138,139,140]. Traffic infractions and a higher frequency of traffic accidents are among the maladaptive effects linked to ADHD [141]. It is also associated with an increased risk of delinquent behaviors and often co-occurs with CD. Research suggests that ADHD is present in 57% of children with CD and often precedes it, following a developmental trajectory in which ADHD symptoms predict the onset of CD [142]. Since it is linked to worse outcomes, including a higher chance of developing ASPD in adulthood, the combination of ADHD and CD is especially concerning [143,144]. CD and several neuropsychiatric disorders, of which ADHD is the most prevalent, have a complex pattern of comorbidity. Due to its frequent overlap with other emotional and behavioral disorders, especially ADHD, this supports earlier findings that place CD within the externalizing spectrum [145,146,147]. Despite having different symptoms, CD and depression frequently co-occur, with CD being linked to internalizing illnesses including anxiety and depression [148]. Importantly, genetic studies suggest a shared genetic liability between ADHD and CD [149]. About 20% of individuals with ADHD fit the criteria for ASPD, demonstrating a close correlation between the two disorders. This development is significantly predicted by CD, particularly if it appears early in life [150]. According to a study by Konstenius et al., women with ADHD were much more likely to use illicit stimulants and have ASPD than women without the disorder. The study also revealed that the prevalence of ADHD in women in prison was high, like rates among male offenders [151]. Consistent with earlier research, ASPD and ADHD have been linked [152,153,154]. Although psychopathy and ADHD share some behavioral characteristics, ADHD is more closely associated with the chronically unstable and antisocial lifestyle aspects of psychopathy, whereas the affective and interpersonal features of psychopathy are not typically associated with ADHD [155,156]. The main area of overlap between psychopathy and ADHD is impulsivity [157]. People with ADHD are more likely to commit violent crimes, including sexual assault, and to display criminal behavior earlier than their peers. However, rather than being deliberate or planned, these behaviors are typically emotional and impulsive. Importantly, early-onset CD appears to mediate progression to antisocial and criminal behavior [156]. The association of ADHD with impulsivity and externalizing tendencies underlines its significant role in behavioral dysregulation and its impact on the development of comorbid disorders. A brief overview of clinical characteristics of ASP, CD, ODD, and ADHD is offered in Table 2.

There is a frequent association between ASPD and substance use disorders (SUDs), in particular alcohol use disorder (AUD), cannabis use disorder (CanUD), and tobacco use disorder (TUD) [158,159].

This may be due to shared characteristics such as impulsivity and behavioral disinhibition. Specific SUD criteria such as ‘risky use’ were strongly associated with ASPD, reflecting its impulsive and reckless nature, while ‘attempts to quit’ were negatively associated, suggesting greater difficulty in reducing substance use among people with ASPD. These findings highlight common mechanisms underlying ASPD-SUD comorbidity and the need for personalized interventions [160].

## 5. Therapeutic Approaches

Recent studies indicate that antisocial behavior can be modified through increasingly effective interventions based on a better understanding of the underlying mechanisms [7,85,161]. Violent adolescents, thanks to the immaturity of this developmental stage, offer greater opportunities for therapeutic intervention than antisocial adults, where these possibilities are often limited. In most cases, violent acts committed in adolescence maintain an interpretable meaning, paving the way for paths of care. Meta-analytic studies outline that the most effective treatment outcomes can be obtained using multimodal and integrated methods, which are based on targeted interventions and oriented to understand the factors that predispose or prevent the onset of criminal behavior. Treatments reduce the probability of relapses by 10 percent on average. Other studies point out that antisocial behavior does not always lead to a criminal life path: about 50 percent of cases evolve positively due to protective factors, such as a favorable family or social environment [162].

The uncertainty surrounding treatment efficacy is often attributed to methodological challenges, including difficulties in obtaining standardized studies, selecting homogeneous comparison groups, ensuring long-term follow-up, and completing intervention programs. In Anglophone countries, there is a predominant preference for a rehabilitation approach centered on cognitive behavioral and systemic models. These approaches aim to enhance the young person’s cognitive, social, and relational skills. Conversely, in Europe, clinicians tend to adopt an integrated model that focuses on understanding the internal conflicts and psychological issues underlying antisocial behavior while simultaneously promoting the recovery of both social and cognitive skills [85].

Since primary prevention is not feasible, secondary prevention programs focus on early interventions designed to identify and address warning signs. These efforts aim to mitigate the risk of developing antisocial personality traits and to prevent the escalation of deviant behaviors. Research highlights that effective preventive strategies require recognizing warning signs within the first five years of life. Key approaches include preschool interventions, parental educational support, early management of disruptive behaviors, and school-based programs [85].

An effective intervention strategy addressing parental difficulties contributing to antisocial behaviors is parent training (PT). PT involves structured sessions designed to improve parental attitudes and relational styles that may negatively affect a child’s behavior. It promotes more positive and functional interactions, creating a supportive environment for children. This approach is particularly beneficial for children exhibiting impulsivity, hyperactivity, and poor self-control, as it equips parents to foster conditions that enhance the child’s ability to self-regulate [163]. Additionally, PT provides parents an opportunity to reflect on their role, gain awareness of their educational choices, and build a sense of efficacy. Ultimately, it helps create a healthier and more supportive environment for the child to thrive [164]. Initial clinical interventions should prioritize identifying environmental variables contributing to problematic behaviors. Psychosocial and psychoeducational interventions should be the first line of action. If these interventions prove ineffective or yield limited outcomes, psychopharmacological therapy can then be considered. Moreover, certain clinical characteristics may render psychotherapy inadvisable: sadistic cruelty towards others, complete absence of remorse, lack of emotional attachments, significantly above-average or below-average intelligence, and intense countertransference fear of being attacked [165].

A double-blind study by Malone et al. on 44 children with CD admitted to a specialized ward for severe aggressive behavior compared the efficacy of lithium treatment versus placebo [166]. Interestingly, approximately 50% of those treated with placebo showed a significant reduction in aggressive behaviors. This finding suggests that the structured containment environment, combined with educational and psychosocial interventions, may be sufficient to alleviate or resolve antisocial behaviors. As a result, many children and adolescents with severe behavioral symptoms might not require pharmacological therapy.

### 5.1. Psychological Interventions

Psychotherapeutic interventions for antisocial behavior in children and adolescents draw from diverse theoretical frameworks, with cognitive behavioral and psycho-dynamic models being among the most influential. Each approach offers unique perspectives and methodologies to address the complex interplay of individual, familial, and environmental factors that contribute to deviant behaviors. Cognitive behavioral interventions focus on identifying and modifying maladaptive thought patterns, improving problem-solving skills, and enhancing family dynamics to foster positive behavioral changes. These approaches are grounded in evidence-based practices, emphasizing structured strategies to achieve measurable outcomes [167]. In contrast, psychodynamic interventions delve into the underlying psychological and emotional conflicts that drive antisocial behavior. By exploring unconscious processes, emotional attachments, and interpersonal dynamics, these approaches aim to provide deeper insights into the root causes of maladaptive behaviors while promoting long-term emotional and relational development [165]. Together, these psychotherapeutic models offer complementary pathways for understanding and managing antisocial behaviors, with each addressing distinct yet interconnected aspects of the individual’s psychological and social world.

### 5.2. Psychotherapeutic Interventions Based on Cognitive Behavioral Models

Developed by Henggeler et al., multisystemic therapy (MST) operates on the theoretical premise that interconnected systems—such as the individual, family, school, peer group, and community—shape reciprocal dynamics and influences. MST emphasizes conducting both assessments and interventions within the child’s real-life contexts, including school, home, and neighborhood. Utilizing evidence-based treatment strategies, MST targets individual factors (e.g., cognitive distortions), familial issues, and external contributors to adolescent violent behavior. A core assumption of MST is that the family is central to achieving sustainable therapeutic outcomes. Consequently, parents play a pivotal role in intervention, with a focus on raising their awareness and addressing any challenges they face [168]. Several studies have documented the effectiveness of MST in reducing recidivism among antisocial adolescents, even in situations characterized by severity, thus representing a valid alternative to prison-type measures [169]. To obtain clinically significant results, it has been demonstrated that it is important to maintain scrupulous adherence to the treatment protocols provided by the model [170,171].

Another effective psychological treatment is Cognitive Problem-Solving Skills Training (PSST). PSST is grounded in the concept that aggression is partially influenced by how individuals perceive and interpret events. The intervention focuses on addressing cognitive distortions in adolescents with deviant behavior, helping them interpret and respond to social situations more adaptively [172,173]. While PSST has shown therapeutic success, its effectiveness diminishes in cases involving very young children, cognitive or academic challenges, or highly problematic family environments [174].

There is a growing emphasis on psychological interventions that focus on family dynamics and parental involvement, recognizing their pivotal role in shaping a child’s behavior and development. One of the most extensively studied and validated interventions, Parent Management Training (PMT), is based on the principle that dysfunctional family interactions significantly contribute to problematic behaviors. The program aims to assist parents in adopting more constructive educational strategies, discouraging deviant behaviors while reinforcing positive ones. Although PMT generally demonstrates lasting effects, its efficacy is limited when families fail to complete the intervention as planned [175]. Another intervention strategy is Functional Family Therapy (FFT), which focuses on the family system as a whole, emphasizing improved communication and resolution of interpersonal conflicts. While it has shown promise in treating young delinquents, its effectiveness has yet to be confirmed through controlled studies. FFT aims to address family dynamics and create a cohesive environment that fosters behavioral improvement [176].

These interventions highlight the importance of addressing both individual and systemic factors in managing antisocial behavior, offering a range of evidence-based approaches tailored to diverse contexts and needs.

### 5.3. Psychotherapeutic Interventions Derived from Psychodynamic Models

Psychodynamic approaches to treating antisocial behavior in adolescents offer a unique perspective by focusing on the unconscious emotional conflicts and psychological processes underlying deviant behaviors. Unlike other methods, psychoanalytic therapy faces challenges in efficacy research due to the inherent difficulty of standardizing its interventions. The primary aim of psychoanalytic therapy is to assist patients in identifying and articulating their emotions, helping them recognize that aggressive reactions often stem from feelings such as anger or humiliation. The ultimate goal is to replace pathological defense mechanisms—which provide only a false sense of control and security—with healthier processes like mentalization and self-regulation [177]. To achieve these outcomes, it is critical to communicate the therapy’s purpose and actively engage the adolescent in the treatment process. Adapting the therapeutic setting to the specific needs of adolescents is crucial for success. This includes employing an “elastic setting”, which emphasizes flexibility, such as reducing session frequency, integrating environmental therapeutic interventions, and replacing traditional psychoanalytic practices like the couch with face-to-face interactions [178,179].

Another therapeutic option involves institutional treatment in settings such as hospital wards, prison facilities, communities, or day centers. A critical aspect of this approach is distinguishing between treatable antisocial patients and psychopathic individuals. In psychopathy, violent acts lack a communicative connection to internal conflicts, making therapeutic interpretation and intervention ineffective. Gabbard and Coyne studied hospitalized antisocial patients and identified predictive factors for positive and negative therapeutic responses. They found that comorbid Axis I diagnoses, such as depression, psychosis, or anxiety, are indicative of treatable conditions and exclude a true psychopathic diagnosis, thus supporting the efficacy of hospital-based treatment. Conversely, patients with histories of arrests, convictions, deceit, or organic brain damage were deemed unsuitable for psychiatric admission [175]. This differentiation is critical for tailoring interventions to the specific characteristics and needs of the patient.

Psychodynamic interventions, while demanding in their application and evaluation, provide valuable insights into the emotional and psychological roots of antisocial behavior and can play a key role in comprehensive treatment strategies.

### 5.4. Pharmacological Treatment Options

The primary goal of pharmacological interventions in managing antisocial and aggressive behaviors is to reduce aggression by modulating the neurobiological systems underlying behavioral activation. Effective pharmacological management in children and adolescents requires adherence to key principles, like reserving medication use for cases where psychosocial interventions have failed, employing standardized assessment tools, such as scales and questionnaires, to monitor progress, and establishing clear treatment objectives, addressing the underlying psychopathological condition contributing to aggression [180,181]. There are important precautions to follow, for example selecting drugs with minimal side effects and prioritizing monotherapy, fully exploring the dosage range and duration of a drug before switching or combining treatments, and monitoring blood levels for specific medications (e.g., antiepileptics, lithium) [161].

Currently, no pharmacological treatments are specifically approved for aggression, and medications are often repurposed from other indications. Among available options, antipsychotics have shown the strongest evidence for reducing aggressive impulsivity. Risperidone, an atypical antipsychotic, demonstrates a favorable tolerability profile and efficacy at doses of 0.5–4 mg/day, typically administered in two doses. Its effects often manifest within the first week of treatment and are sustained over time. However, discontinuation leads to a relapse of aggressive behaviors in approximately 75% of patients [182,183,184]. Haloperidol, a first-generation antipsychotic, has also proven effective at doses of 1–6 mg/day. Its use is limited by the high risk of dose-dependent side effects, including neuroleptic malignant syndrome, making it a second-line choice for aggression management [185]. Limited data exist on the efficacy of other antipsychotics, such as clozapine, aripiprazole, and olanzapine, in childhood aggression.

If a diagnosis of ADHD is formulated during the clinical assessment, a psychostimulant prescription may benefit the adolescent with violent behavior. Methylphenidate is effective in managing aggression in children with ADHD, provided the aggression is not linked to psychotic or manic disorders. Careful psychopathological evaluation is critical, as inappropriate use may exacerbate aggression [186].

Other therapeutic strategies may involve different classes of psychoactive drugs. Mood stabilizers have shown limited efficacy in managing aggression. Two placebo-controlled studies highlighted the role of lithium in reducing violent behaviors in hospitalized children with severe conduct disorders. Effective dosages ranged from 900 to 2100 mg/day, though high plasma levels (>1.1 mEq/L) showed little additional benefit [187,188]. In less severe cases or in outpatient settings, lithium demonstrated no significant advantage over placebo [189]. Valproate has shown some promise in reducing aggression, as evidenced by small placebo-controlled studies [190,191,192]. There is also a possible therapeutic effect of serotonergic drugs (such as SSRIs) even if they should be used with caution, since in some situations these can cause the onset of irritability, anxiety, and worsening of behavior [193].

There is limited evidence supporting the efficacy of pharmacological or psychological interventions for adults with ASPD. A study found that an intervention with clinical efficacy in adults is Mentalization-Based Treatment (MBT-ASPD), which focuses on the mental and relational processes underlying the disorder [194]. Also carried out in group contexts, MBT-ASPD addresses vulnerabilities related to mentalization capacity and attachment patterns and promotes the creation of a sense of collective belonging. Studies have shown partial benefits of psychological interventions in adults with ASPD and substance use disorders. While these interventions have been effective in managing substance dependence, they have shown little impact on antisocial behavior [195,196].

Pharmacological approaches remain a complementary option in adults, particularly when comorbidities such as anxiety, depression, or substance use disorders influence behavioral patterns.

## 6. Focus on Violent Behavior and Forensic Evaluation

Violent behavior in juveniles represents a significant challenge for mental health professionals, legal systems, and society. As adolescents navigate a critical developmental phase marked by biological, psychological, and social changes, some exhibit patterns of aggression and antisocial conduct that may lead to criminal activity. Forensic evaluation plays a pivotal role in understanding the underlying causes of violent behavior, assessing risk, and guiding judicial decisions. This process not only addresses individual accountability but also emphasizes rehabilitation and societal protection. Violent behavior in juveniles encompasses physical aggression, destruction of property, and, in severe cases, acts like assault or homicide. Such behaviors may arise from impulsive reactions, emotional dysregulation, or premeditated intent to harm [197,198,199].

Adolescents are in a unique phase of neurodevelopment where emotional and cognitive systems are still maturing. The prefrontal cortex, responsible for impulse control and decision-making, develops slower than the limbic system, which drives emotional responses. This mismatch can result in heightened impulsivity and poor judgment, contributing to violent acts [96].

What is classified from the age of 18 onward as ASPD has its roots in a pervasive pattern of disregard for and violation of others’ rights already present in early adolescence and young adulthood, diagnosed as CD. These psychopathological patterns represent significant areas of interest in the assessment of antisocial behavior, as well as violent and delinquent behaviors in young adulthood. The terms antisocial and psychopathy are often used interchangeably. While conduct disorder and ASPD are defined solely by behavioral criteria (such as impulsivity and often aggression), psychopathy requires consideration of emotional and personality traits, such as the absence of anxiety or any other symptom attributable to a neurotic or psychotic condition [91,102].

In psychopathological assessments conducted in both clinical and forensic contexts, it is important to consider a range of variables (genetic, social, and environmental) which contribute in various but significant ways to shaping the developmental trajectory of antisocial behavior. It is therefore necessary to analyze the potential developmental pathway that a child follows when developing CD. As proposed by Frick, a central aspect of the assessment is the age at which deviant behavior begins. Children who exhibit such behavioral patterns early in childhood, with a gradual increase in intensity and severity, tend to engage in more aggressive and deviant behavior compared to those whose onset coincides with puberty [200]. In the first group, the likelihood that CD will evolve into ASPD over the years is higher, with fewer possibilities for intervention than in the second group. Another factor to consider is the presence or absence of callous(insensitive)/unemotional (CU) trait, characterized by a lack of empathy and remorse. It has been shown that the presence of this trait significantly correlates with more aggressive behavior patterns, a tendency to engage in dangerous and deviant activities, and reduced sensitivity to punishment [200]. Focusing on these variables also guides the choice of more effective intervention strategies: children exhibiting a predominance of the CU trait benefit more from psychological interventions aimed at fostering empathy, whereas those without or with reduced levels of this trait benefit more from approaches focused on impulse control.

Forensic evaluations aim to provide a comprehensive understanding of a juvenile’s behavior in relation to their legal and social circumstances. This process integrates clinical assessment, legal principles, and rehabilitative goals. The objectives of forensic evaluation are to determine accountability, defined as the juvenile’s ability to understand the consequences of their actions and distinguish right from wrong, to evaluate the risk of future violent behavior and reoffending and to provide recommendations for treatment, educational support, or therapeutic interventions. The foundation of forensic assessment includes a detailed clinical interview, exploring the adolescent’s developmental history, family environment, emotional experiences, and patterns of behavior. It also involves gathering collateral information from caregivers, teachers, and legal records. It is pivotal to remember that delinquency is not an event but must be considered as a process that starts with precocious conduct problems, such as conflicts in school or family, and antisocial behaviors [201,202].

In addition to the clinical interview, a comprehensive evaluation requires further information that can be obtained through standardized psychometric assessment tests and projective tests. These tools provide a broader categorization of the subject, quantify the risk factors for recidivism, and identify psychopathic traits, such as a lack of remorse or empathy. It is important to remember that no single variable or psychodiagnostic test is sufficient on its own to formulate a diagnosis. Instead, it is the convergence or divergence of multiple parameters, combined with the clinical interview, that allows for a more accurate clinical diagnosis. Moreover, the administration of neuropsychological tests, including assessments of executive functioning, impulse control, and emotional regulation, is crucial, given their role in violent behavior. Forensic evaluations should also include risk and protective factors analysis, evaluating external factors like family support, school engagement, or substance abuse helping contextualize behavior and predict outcomes, and legal competency evaluation, determining whether the juvenile can understand legal proceedings and participate effectively in their defense [203].

Initial test batteries should evaluate broad psychological dynamics, followed by the use of more specific psychodiagnostic tools selected on the base of diagnostic hypotheses to be confirmed. The most common tests used to gain information about the individual’s basic organization and personality structure are the Minnesota Multiphasic Personality Inventory-2 (MMPI-2) and the Millon Clinical Multiaxial Inventory (MCMI).

The MMPI-2 is the most widely used and studied psychodiagnostic tool, providing a description of personality and psychopathological dimension through various clinical, content, and supplementary scales. It includes about 50 items assessing deviant behaviors, cynicism, and egocentrism. A specific scale for psychopathic deviation (Scale 4, Pd), combined with the hypomania scale (Scale 9, Ma), is often elevated in psychopathic profiles, serving as excellent predictors of social deviance. Additionally, the content scale for depression (Dep) is frequently elevated, likely reflecting the conditions of incarceration and detention. Empirical studies show that Scale 4 (Pd) positively correlates with the second factor of the PCL-R (social deviance) but not with the first factor (affective interpersonal style), emphasizing the behavioral component of psychopathic personality. The Antisocial Practices Scale (APS), a content scale composed of 22 items, is particularly relevant for studying antisocial personality. It investigates illegal behaviors, psychoactive substance use, legal violations, and school conduct problems [204,205,206].

The MCMI is frequently used for personality disorder evaluation. Based on Millon’s model, it includes 175 items assessing 14 personality disorder scales and 10 scales evaluating mood disorders, anxiety, and thought disorders. Among these, scale 6A specifically assesses antisocial traits, exploring impulsivity, responsibility, and current or past conduct disorders. Elevations in scale 6A and the narcissism scale are characteristic of severe antisocial profiles [207].

Among the most widely accepted and scientifically validated tools is the Psychopathy Checklist (PCL), which has an adapted version for youth aged 12 to 18, the PCL-Youth Version (PCL-YV). The youth version comprises 20 items and is completed after the clinical interview and an extensive collection of anamneses, involving both the subject and their family. Forensic psychiatrists should use all available clinical, forensic, and anamnesis material while administering this checklist. Each item, except for items 11 and 17, is categorized into four factors: interpersonal, affective, behavioral, and antisocial. Research has shown that the PCL score can predict antisocial behavior, psychopathy indicators, and attachment styles, making it the most commonly used tool for psychopathy assessment in forensic and correctional settings. Supporting its validity, studies demonstrate a correlation between the total PCL score and Scales 4 and 9 of the MMPI-2 [208,209].

The evaluation of violent juveniles has several critical implications for legal and therapeutic outcomes. It should evaluate the risk of recidivism, stratifying this risk and guide interventions that target specific factors, such as impulse control or environmental triggers. One of the most contentious decisions in juvenile cases involves determining whether the offender should be tried as an adult. These evaluations inform this decision by assessing the adolescent’s developmental maturity, culpability, and amenability to rehabilitation [203].

Juvenile justice systems prioritize rehabilitation over punishment: in this scenario, forensic evaluations provide a roadmap for therapeutic interventions, such as cognitive behavioral therapy, anger management programs, or family therapy, while highlighting the importance of community-based support systems [202,210]. By identifying the roots of violent behavior, assessing risk, and guiding rehabilitative measures, forensic psychiatry plays a pivotal role in shaping outcomes for juveniles and society. A balanced approach that considers developmental immaturity, environmental influences, and potential for rehabilitation can help these individuals reintegrate into society as productive and law-abiding citizens.

## 7. Future Directions for Preventing Antisocial Behavior and Conclusions

Antisocial behavior in children and adolescents presents a complex interplay of biological, psychological, and social factors, posing challenges for early identification, effective intervention, and long-term prevention. This narrative review has emphasized the developmental trajectory of antisocial behaviors, from early conduct problems to the more entrenched patterns observed in ASPD. Key findings highlight the role of genetic predispositions, neurobiological dysfunctions, and environmental adversities in shaping antisocial tendencies. Disruptions in brain regions such as the prefrontal cortex, amygdala, and limbic structures, coupled with hormonal and neurotransmitter imbalances, contribute to emotional dysregulation, poor impulse control, and deficits in empathy and social cognition. These impairments are compounded by external factors, such as adverse family dynamics, socioeconomic disadvantages, and exposure to deviant peer groups, which further entrench antisocial behaviors.

Despite advances in our understanding of the etiology and progression of antisocial behavior, significant gaps remain in the effectiveness and accessibility of therapeutic interventions. Behavioral and psychosocial approaches, including cognitive behavioral therapy (CBT), multisystemic therapy (MST), and family-focused interventions, have demonstrated potential. However, their success is often limited by implementation challenges, such as the heterogeneity of patient profiles, variability in access to care, and high rates of treatment discontinuation. Pharmacological treatments offer some promise, particularly in managing comorbid conditions like ADHD or mood disorders, but their application is constrained by concerns about efficacy, side effects, and the lack of tailored options for younger populations.

The forensic implications of antisocial behavior demand particular attention. Adolescents with severe conduct problems often come into contact with the juvenile justice system, where the focus tends to shift from rehabilitation to punishment. This approach risks exacerbating underlying issues, as incarceration can reinforce antisocial tendencies through exposure to criminal subcultures and further social marginalization. Forensic assessments play a pivotal role in understanding the developmental, neurobiological, and psychosocial factors underlying violent and antisocial behaviors. Risk assessments for recidivism, evaluations of culpability, and the identification of mitigating factors are critical for shaping judicial decisions. Furthermore, forensic evaluations can guide the implementation of rehabilitation programs tailored to the needs of young offenders, emphasizing therapeutic over punitive approaches.

Looking ahead, future research must adopt a multidisciplinary and integrative perspective to address these challenges comprehensively. Longitudinal studies are essential to delineate the causal pathways linking early-life adversities, neurobiological vulnerabilities, and environmental influences on antisocial outcomes. These studies should aim to identify biomarkers—such as neuroimaging patterns, genetic variants, or hormonal profiles—that can facilitate early detection and risk stratification. Advances in neuroimaging, particularly functional MRI and connectomics, offer opportunities to deepen our understanding of the neural circuits implicated in antisocial behavior, including those governing emotion regulation, moral reasoning, and social cognition [211].

Intervention research must prioritize the customization of treatment protocols based on individual profiles, including gender-specific presentations, neurocognitive strengths and weaknesses, and the presence of comorbid psychiatric conditions. For example, interventions targeting emotional dysregulation and impulsivity could benefit from integrating mindfulness-based approaches, biofeedback, and cognitive training alongside traditional therapies. Similarly, forensic-specific interventions, such as restorative justice programs and violence prevention initiatives, should be further evaluated for their ability to reduce recidivism and promote rehabilitation among youth with antisocial tendencies.

From a public health perspective, preventative strategies must focus on addressing the root causes of antisocial behavior. Universal screening for conduct problems, early intervention programs targeting at-risk families, and school-based initiatives to enhance emotional regulation and social skills are critical components. Policy-driven measures to reduce socioeconomic inequalities and improve access to mental health services can mitigate many of the risk factors associated with antisocial behavior. The integration of mental health and forensic services within community settings can also bridge gaps in care and provide a more holistic response to youth exhibiting antisocial tendencies [194].

The forensic aspect of antisocial behavior also underscores the need for reforms in the juvenile justice system. Collaborative efforts among mental health professionals, legal experts, and social workers can ensure that judicial processes are informed by a nuanced understanding of developmental psychopathology. Evidence-based risk assessment tools should be integrated into judicial decision-making to support rehabilitative outcomes, particularly for adolescents with significant neuropsychological or emotional impairments.

In conclusion, addressing antisocial behavior in youth requires a sustained commitment to multidisciplinary research, innovative intervention design, and systemic reforms in both healthcare and legal frameworks. By deepening our understanding of the biological and social underpinnings of antisocial tendencies and translating this knowledge into actionable strategies, we can reduce the burden of this disorder and foster healthier developmental trajectories. Strengthening the links between science, policy, and practice will be critical in creating a society that prioritizes early prevention, effective treatment, and compassionate rehabilitation for individuals at risk.

## Figures and Tables

**Table 1:** Factors influencing the developmental trajectory of antisocial behavior.

Category	Risk Factors	Protective Factors
Individual factors	- Temperament issues (irritability, poor emotional regulation)	- Adaptive social skills and emotional intelligence
	- Behavioral inhibition (e.g., apprehension, anxiety, shyness)	- Behavioral activation (e.g., curiosity, sensation-seeking in appropriate contexts)
	- Intellectual and neuropsychological difficulties (low Intelligence Quotient, executive dysfunction)	- Positive problem-solving skills
	- Early substance use	- Abstinence from substance use
	- Social withdrawal	
	- Impulsivity, hyperactivity	
Family factors	- Socioeconomic disadvantages (poverty, unemployment)	- Stable family bonds
	- Low parental education	- Supportive and consistent parenting
	- Parental antisocial behaviors or psychopathology (e.g., depression, ASPD)	- Positive role models within the family
	- Abuse, neglect, or inconsistent parenting	- Family environment fostering open communication and emotional support
	- Parental substance use	- Absence of intergenerational trauma
School factors	- Poor academic performance	- High academic achievement and motivation
	- Lack of interest or engagement in school activities	- Positive relationships with teachers and peers
	- Inefficient or unsupportive educational institutions	- Access to schools promoting inclusive and individualized learning
Peer group factors	- Association with deviant peers	- Friendships with prosocial peers
	- Isolation due to peer rejection	- Social inclusion and community support
Community factors	- Exposure to violent or unsafe environments	- Availability of resources (e.g., recreational programs, mentorship opportunities)
	- Lack of community support	- Community initiatives fostering social cohesion and collective efficacy

**Table 2:** Overview of clinical characteristics of ASPD, CD, ODD, and ADHD in pediatric/adolescent patients.

Disorder	Key Features	Onset	Comorbidities	Risk Factors	Long-Term Outcomes
Antisocial Personality Disorder (ASPD)	Pervasive disregard for others’ rights, deceitfulness, impulsivity, aggression, lack of remorse	Adolescence (must have a history of CD before 15 years old)	Substance use disorders, depression, anxiety, ADHD	Genetic predisposition, childhood trauma, low socioeconomic status, parental ASPD	High risk of criminal behavior, incarceration, substance abuse, interpersonal issues
Conduct Disorder (CD)	Aggression towards people/animals, property destruction, deceitfulness/theft, serious rule violations	Childhood or adolescence	ADHD, depression, anxiety, substance use disorders	Harsh/inconsistent parenting, peer delinquency, exposure to violence, neurobiological deficits	Risk of progressing to ASPD, academic failure, legal issues
Oppositional Defiant Disorder (ODD)	Persistent anger, argumentative/defiant behavior, vindictiveness, refusal to comply with authority	Early childhood	ADHD, mood disorders, learning disorders	Parental conflict, poor emotional regulation, early childhood trauma, inconsistent discipline	Risk of developing CD, social difficulties, academic struggles
Attention-Deficit/Hyperactivity Disorder (ADHD)	Inattention, hyperactivity, impulsivity, executive dysfunction, emotional dysregulation	Early childhood	CD, ODD, mood disorders, substance use disorders	Genetic factors, prenatal exposure to toxins, early childhood adversity	Academic and occupational difficulties, increased risk of substance abuse, impulsive decision-making

## Data Availability

No new data were created for this study.
